# Suppressive Effect of the n-Hexane Extract of* Litsea japonica* Fruit Flesh on Monosodium-Iodoacetate-Induced Osteoarthritis in Rats

**DOI:** 10.1155/2017/1791403

**Published:** 2017-08-22

**Authors:** Seung-Hyung Kim, Hye-Jin Choi, Won-Kyung Yang, Ji-Eun Lee, Ju-Hyun Cho, In-Jae Park, Sunyoung Park, Bo-Kyung Park, Mirim Jin

**Affiliations:** ^1^Institute of Traditional Medicine and Bioscience, Daejeon University, Daejeon 34520, Republic of Korea; ^2^Laboratory of Molecular Medicine, College of Korean Medicine, Daejeon University, Daejeon 34520, Republic of Korea; ^3^Hurum Central Research Institute, Hurum Co., Ltd., Chungcheongbuk-do, Cheongju-si 28116, Republic of Korea; ^4^College of Medicine, Gachon University, Incheon 21999, Republic of Korea; ^5^KM Convergence Research Division, Korea Institute of Oriental Medicine, Daejeon 34054, Republic of Korea

## Abstract

We examined the antiosteoarthritic effect of the n-hexane extract of* Litsea japonica* fruit flesh (LJF-HE) in a rat model of monosodium-iodoacetate- (MIA-) induced osteoarthritis. LJF-HE significantly reduced the difference in weight-bearing capabilities of the hind paws between healthy and MIA-treated rats. Histological examination of the knee joints indicated that LJF-HE suppressed cartilage and bone destruction. Additionally, there were decreases in the expression of matrix metalloproteinase-2 and metalloproteinase-9 and cyclooxygenase-2 in the joints. The serum levels of deoxypyridinoline (DPD) and osteocalcin, which are markers of bone metabolism, also decreased. Furthermore, LJF-HE significantly suppressed infiltration of inflammatory cells into the synovium and inhibited the expression of proinflammatory cytokines such as tumor necrosis factor- (TNF-) *α*, interleukin- (IL-) 1, and IL-6 in the joints and serum. The serum levels of leukotriene B4 and lipoxygenase were also significantly lowered by LJF-HE. Finally, LJF-HE inhibited the production of nitric oxide, prostaglandin E2, IL-6, and TNF-*α* in lipopolysaccharide-activated macrophages, which might be associated with inhibited phosphorylation of p38 mitogen-activated protein kinase and c-Jun N-terminal kinase. Our data suggest that LJF-HE has an anti-inflammatory effect and may have potential as an antiosteoarthritic agent.

## 1. Introduction

Osteoarthritis (OA) is a degenerative joint disease in the aging population that can cause disability. The functional limitation in the affected joint, which is caused by progressive degradation of cartilage and subchondral tissue remodeling, leads to bone deformity [1]. It is reported that metabolic interactions between cartilage and bones play pivotal roles in the pathogenesis of OA [2]. During OA progression, activated osteoblasts induce the production of matrix metalloproteinases (MMPs) by chondrocytes, which leads to cartilage degradation [3]. Subchondral bone osteoblasts produce proinflammatory cytokines such as interleukin- (IL-) 1, IL-6, and tumor necrosis factor-*α* (TNF-*α*). These trigger osteoclast activity, which leads to bone resorption [4] and an imbalance in bone metabolism. As a result, catabolism is favored while there is a decrease in anabolism. This leads to bone remodeling. Activation of cyclooxygenase- (COX-) 2 and lipoxygenase (LOX) results in the production of inflammatory mediators such as prostaglandins and leukotrienes from arachidonic acid, which also contributes to the development and progression of OA [5].

*Litsea japonica (L. japonica) Jussieu* (Lauraceae) is grown in the southern areas of Korea and Japan and used as a herbal medicine. It has been reported that the fruit of this plant has antioxidant [6], anti-inflammatory [7], and antidiabetic effects [8]. It contains various anti-inflammatory and analgesic phytochemicals including hamabiwalactone A and hamabiwalactone B (A) [9].

We have previously reported that the ethanolic extract of this fruit has a significant antiosteoarthritic effect, which is exerted via suppression of cartilage degradation and subchondral bone deformation [10]. One of the initial steps in the study was elucidation of the active compound(s) in the fruit. We fractionated the total extract of the fruit (flesh only) using organic solvents with various polarities and found that the hexane fraction (LJF-HE) has a beneficial effect on joint health. In the present study, we evaluated the in vitro and in vivo antiosteoarthritic and anti-inflammatory effects of LJF-HE.

## 2. Materials and Methods

### 2.1. Preparation of* L. japonica* Fruit n-Hexane Extract (LJF-HE)

*L. japonica *fruits were collected from Jeju Island in Korea. The samples were stored in the natural products library of Hurum Central Research Institute (specimen number HR-1506003). The freeze-dried fruit (1,000 g) was extracted once with n-hexane for 24 h at room temperature and concentrated under reduced pressure to obtain LJF-HE. The yield was approximately 30.6% (w/w).

### 2.2. High-Performance Liquid Chromatography (HPLC) Analysis

Using hamabiwalactone B as a surrogate marker, LJF-HE was subjected to HPLC using an Alliance Waters 2695 separation module coupled to a Waters 2998 photodiode array detector. A Cadenza CD-C18 column (4.6 mm × 150 mm; particle size, 5 *μ*m; Imtakt, Portland, OR, USA) was used for chromatographic separation. The column was placed in a column oven set at 30°C. The mobile phase consisted of 0.5% acetic acid (A) and acetonitrile (B) and was set at a flow rate of 1.0 mL/min. The ratios of A and B were varied over the run time (gradient elution) as shown in Table 1. The injection volume was set at 10 *μ*L, and UV detection was done at 254 nm. Quantification of hamabiwalactone B in LJF-HE was done using the external standard method. Standard stock solutions (12.5, 25, 50, 100, and 200 *μ*g/mL) of pure hamabiwalactone B (NPC BioTechnology Inc., Daejeon, Korea) were prepared for the analysis.

### 2.3. In Vivo Study: Anti-Osteoarthritic Activity of LJF-HE

#### 2.3.1. Animals

Male Sprague-Dawley (SD) rats (7 weeks old) were purchased from Orient Bio Inc. (Seongnam, Korea) and housed under the following conditions: 12/12-h light/dark cycle; temperature, 22 ± 2°C; and humidity, 55 ± 15%. The rats were provided with laboratory diet and water ad libitum. All procedures performed on the animals were according to the Guide for the Care and Use of Laboratory Animals (National Institutes of Health, Bethesda, MD, USA). The study protocol was approved by the Institutional Animal Care and Use Committee of Daejeon University (Daejeon, Korea) (Approval number DJUARB2016-006).

#### 2.3.2. Induction of OA

The animals were randomized and grouped (*n* = 7 per group) before the initiation of the study. Monosodium-iodoacetate (MIA) (3 mg/50 *μ*L in 0.9% saline) was directly injected into the intra-articular space of the right knee under anesthesia with a mixture of ketamine and xylazine. The rats were then randomly divided into four groups: saline group (no MIA injection), control group (injected with MIA), indomethacin (IM) group (treated with 1 mg/kg IM), and LJF-HE group (treated with 100, 50, or 25 mg/kg of LJF-HE and injected with MIA). The rats were orally administered LJF-HE or IM for a week before injection with MIA. The doses of LJF-HE and IM used in this study were based on those used in previous studies [11].

#### 2.3.3. Measurement of Hind Paw Weight-Bearing Distribution

After OA induction, the original balance in the weight-bearing capabilities of the hind paws was disrupted. An incapacitance tester (Linton instrumentation, Norfolk, UK) was used to evaluate changes in weight-bearing tolerance. The rats were carefully placed in the measuring chamber, after which the weight-bearing force exerted by each hind limb was averaged over a 3 s period. The weight distribution ratio was calculated using the following equation: [weight in right hind limb/(weight in right hind limb + weight in left hind limb)] × 100.

#### 2.3.4. Measurements of Gelatinase Level and COX-2 Activity in Articular Cartilage

Articular cartilage was harvested 21 days after the MIA injection was administered. Each joint cartilage tissue was homogenized in lysis buffer (50 mM Tris-HCl, 150 mM NaCl, 1% Nonidet P-40, 0.1% sodium dodecyl sulfate (SDS), 0.1% deoxycholic acid, 2 *μ*g/mL leupeptin, 2 *μ*g/mL aprotinin, and 1 mM PMSF; pH. 7.4) for 3 h at 4°C. The homogenate was centrifuged at 15,000 ×g for 15 min at 4°C. The supernatant was removed and 20–30 *μ*g of it was diluted in Zymogram Sample Buffer (161–0764; Bio-Rad Laboratories, Inc., Hercules, CA, USA). The diluted samples were stored at −70°C until analysis. The levels of gelatinase A (MMP-2), gelatinase B (MMP-9), and COX-2 in the samples were measured using enzyme-linked immunosorbent assay (ELISA) kits (R&D Systems, Minneapolis, MN, USA) according to the manufacturer's instructions.

#### 2.3.5. Measurements of Inflammatory Cytokines and OA Mediators

Blood samples were centrifuged at 1,500 ×g for 10 min at 4°C, after which serum was collected and stored at −70°C until analysis. Urine was collected after keeping rats in metabolic cages and giving them free access to water but not food for 16 h. The serum levels of IL-1*β*, IL-6, leukotriene B4 (LTB4), and osteocalcin, and the urine levels of deoxypyridinoline (DPD) were measured using ELISA kits from R&D Systems according to the manufacturer's instructions.

#### 2.3.6. Real-Time Quantitative Reverse Transcription Polymerase Chain Reaction (PCR) Analysis

Total RNA was extracted from knee joint tissue using TRI reagent® (Sigma-Aldrich, St. Louis, MO, USA), reverse-transcribed into cDNA, and PCR-amplified using a one-step RT-PCR kit with SYBR green (Applied Biosystems®, Grand Island, NY, USA). Real-time quantitative PCR was performed using a real-time PCR system (7500, Applied Biosystems). The primer sequences and the probe sequence are shown in Table 2. Aliquots of sample cDNAs and an equal amount of glyceraldehyde 3-phosphate dehydrogenase (GAPDH) cDNA were amplified with a TaqMan® Universal PCR master mixture containing DNA polymerase (Applied Biosystems, Foster, CA, USA) according to the manufacturer's instructions. The PCR conditions were 2 min at 50°C, 10 min at 94°C, 15 s at 95°C, and 1 min at 60°C for 40 cycles. The concentration of the target gene was determined using the comparative Ct (threshold cycle number at cross-point between amplification plot and threshold) method (Table 2).

#### 2.3.7. Histopathological Analysis

Tissue specimens from the knee joints of the rats were removed, fixed in 10% formalin, embedded in paraffin, and serially sectioned at 7 *μ*m. The tissue sections were then stained with hematoxylin and eosin (H&E) or Safranin O-fast green. Histological changes were examined by light microscopy (Olympus CX31/BX51; Olympus Optical Co., Tokyo, Japan) and photographed (Olympus DP70).

### 2.4. In Vitro Anti-Inflammatory Activity

#### 2.4.1. Cell Culture and Sample Treatment

Raw 264.7 cells were obtained from the American Type Culture Collection (Manassas, VA, USA) and grown in Dulbecco's modified Eagle's medium (DMEM) containing 1% antibiotics and 5.5% fetal bovine serum. The cells were incubated in a humidified atmosphere of 5% CO_2_ at 37°C. The medium was replaced with fresh DMEM and the cells were stimulated with 1 *μ*g/mL lipopolysaccharide (LPS, Sigma-Aldrich) in the presence or absence of LJF-HE (1–6 *μ*g/mL) for 24 h.

#### 2.4.2. Determination of Nitric Oxide (NO), Prostaglandin E2 (PGE2), TNF-*α*, and IL-6 Production in the Cells

Cells were treated with LJF-HE and then stimulated with LPS for 24 h. NO production was analyzed by measuring nitrite levels using an NO detection kit (iNtRON Biotechnology, Seongnam, Korea) according to the manufacturer's instructions. Secretion of the inflammatory cytokines PGE_2_, TNF-*α*, and IL-6 was determined using an ELISA kit (R&D systems) according to the manufacturer's instructions.

#### 2.4.3. Western Blot Analysis

Whole cell lysates were obtained from cells lysed in 100 *μ*L of radioimmunoprecipitation assay buffer (Sigma-Aldrich) containing protease and phosphatase inhibitors (Sigma-Aldrich). Cytoplasmic and nuclear extracts were obtained using a cytoplasmic and nuclear extract kit (Active Motif, Carlsbad, CA, USA) according to the manufacturer's instructions. The concentration of protein in each sample was determined by the bicinchoninic acid assay. Cell lysates were separated by SDS-polyacrylamide gel electrophoresis and transferred onto polyvinylidene difluoride membranes (Bio-Rad Laboratories, Inc.). The membranes were then incubated with primary antibodies (extracellular signal-regulated kinase (ERK)/p-ERK, p38/p-p38, c-Jun N-terminal kinase (JNK)/p-JNK, nuclear factor kappa B (NF-*κ*B) p65; 1 : 1,000; Cell Signaling Technology, Inc., Beverly, MA, USA). Immune complexes were visualized using an enhanced chemiluminescence detection system (Pierce, Rockford, IL, USA) and a Bio-Rad electrophoresis image analyzer. The densitometric value for each band was determined using ImageJ software (National Institutes of Health).

### 2.5. Statistical Analysis

Data were analyzed by one-way analysis of variance followed by Student's two-tailed *t*-test for comparison between two groups; however, Dunnett's test was used when the data involved three or more groups. Results have been expressed as mean (standard error of the mean). *p* values < 0.05 were considered statistically significant.

## 3. Results

### 3.1. HPLC Analysis of LJF-HE

**Figure 1:**
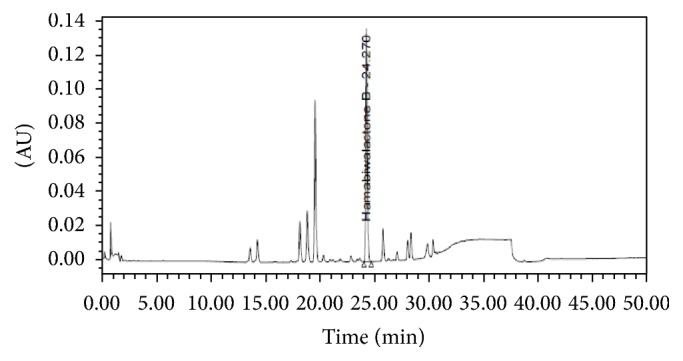
*Preparation of LJF-HE*. Chromatogram of LJF-HE.

Hamabiwalactone B was identified by comparing its retention time and UV spectrum with those of the commercial standard. As is shown in Figure 1, we measured the concentration of hamabiwalactone B as a surrogate marker in LJF-HE. Our results showed that the amount of hamabiwalactone B in the extract was 15.23 ± 0.057 mg/g (Figure 1).

### 3.2. Effect of LJF-HE on Hind Paw Weight-Bearing Distribution in Rats with MIA-Induced OA

**Figure 2:**
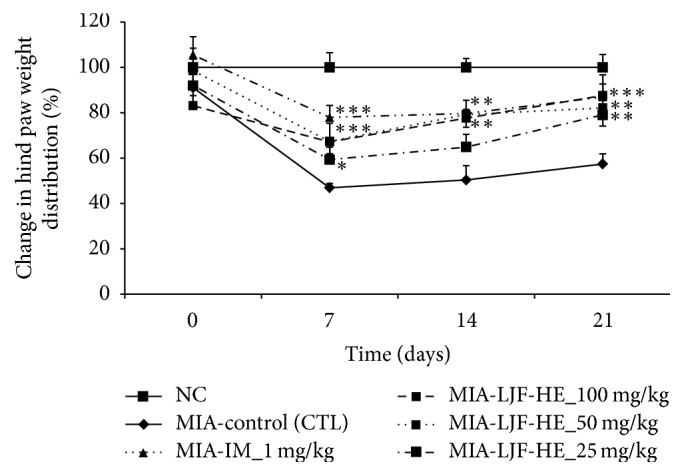
*Changes in hind paw weight-bearing distribution in rats with MIA-induced OA*. Changes in hind paw weight-bearing distribution was measured once a week for three weeks after the injection with MIA. *∗* indicates *p* < 0.05, *∗∗* indicates *p* < 0.01, and *∗∗∗* indicates *p* < 0.001 when compared to the control group.

We evaluated the weight-bearing capabilities of the hind paws using an incapacitance tester at days 0, 7, 14, and 21. The ratio of weight distribution between the right (MIA-injected) and left (healthy) limbs was used to assess the progression of OA. The ratio for the control group was significantly lower than that for the normal group at day 7, and this difference was maintained until day 21. However, at day 7, the ratios for the LJF-HE- and IM-treated groups were slightly lower than the ratio for the control group. Moreover, LJF-HE and IM had dose-dependent effects on the ratio (Figure 2). In particular, the effect of LJF-HE at a dose of 100 mg/kg was prominent and the balance between the two hind legs nearly returned to the normal level at day 21. These results suggest that LJF-HE possibly suppresses MIA-induced OA.

### 3.3. Effects of LJF-HE on Cartilage Degradation and Bone Resorption

**Figure 3:**
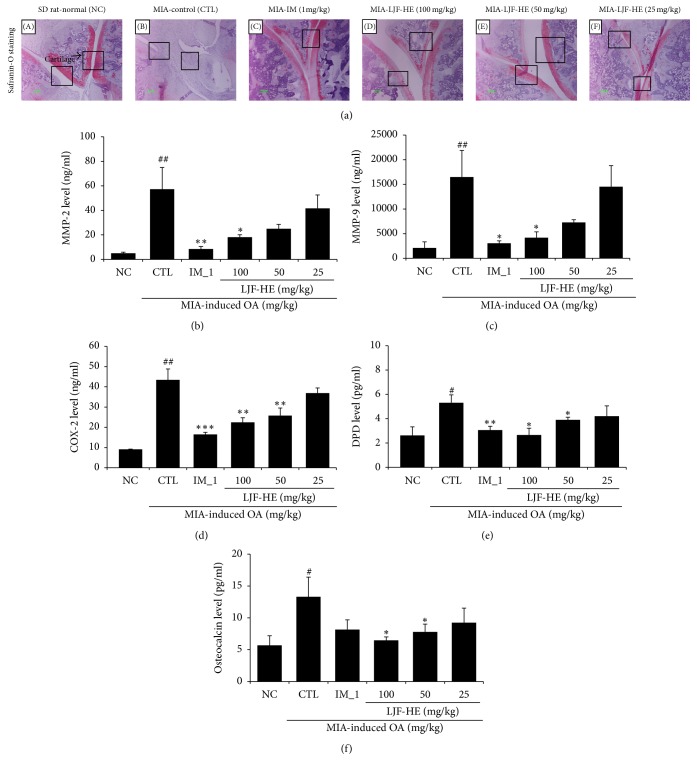
*Histopathological features of the knee joint tissues of rats with MIA-induced OA*. (a) Representative histological analysis (Safranin O staining) of the knee joint in MIA-induced OA. (b–d) The levels of gelatinase A (MMP-2), gelatinase B (MMP-9), and COX-2 in the femur joint were measured by ELISA. The levels of (e) DPD in urine and (f) osteocalcin in serum were measured by ELISA. # indicates *p* < 0.05 and ## indicates *p* < 0.01 when compared to saline group (NC). *∗* indicates *p* < 0.05, *∗∗* indicates *p* < 0.01, and *∗∗∗* indicates *p* < 0.001 when compared to the control group (CTL).

Effects of LJF-HE on cartilage and bone were evaluated by histological examination. The Safranin O staining showed that proteoglycan was well preserved in the joints of the naïve (Figure 3(a): (A)) and LJF-HE-treated (Figure 3(a): (C)) rats, but not in the joints of the control rats, which resulted in complete loss of proteoglycan (no red color) and bone resorption (red color). Consistent with the histological data, the production of MMP-2 and MMP-9, which are cartilage-degrading enzymes, markedly increased in the MIA-treated control group. However, LJF-HE significantly reduced the production of the enzymes in a dose-dependent manner (Figures 3(b) and 3(c)). The production of COX-2 was also significantly inhibited by LJF-HE. The effects of LJF-HE at a dose of 100 mg/kg were comparable to those of IM.

To further investigate the effects of LJF-HE on bone metabolism, we examined the levels of DPD, a marker of bone resorption [12], and osteocalcin, a biochemical marker of bone formation [13]. The results indicated significant increases in urine DPD and serum osteocalcin levels in the MIA-treated rats, which presented an imbalance in bone metabolism [14]. However, LJF-HE significantly suppressed the MIA-induced increase in the levels of the markers. These data suggest that LJF-HE might have suppressive effects on cartilage degradation and bone resorption in MIA-induced OA.

### 3.4. In Vivo Effects of LJF-HE on MIA-Induced Inflammation

**Figure 4:**
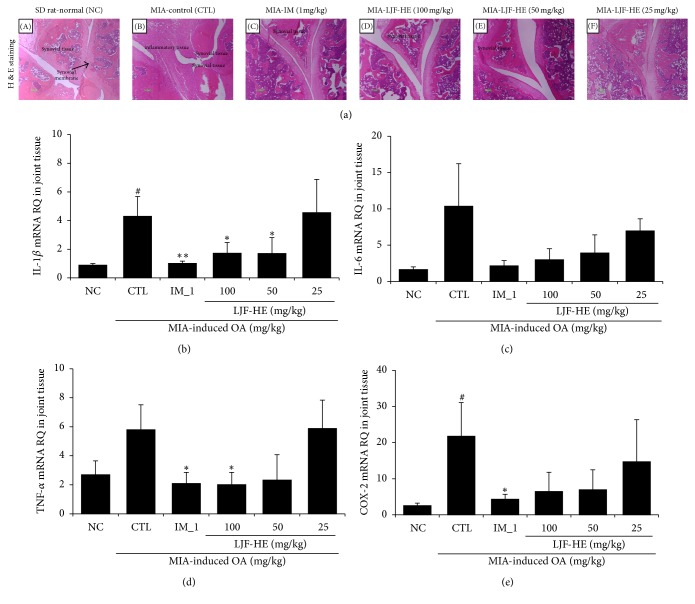
*Effects of LJF-HE on the mRNA expression levels of cytokines in the joint tissues of rats with MIA-induced OA*. (a) Representative histological analysis (H&E staining) of the knee joint in MIA-induced OA. (b–e) The mRNA expression levels of IL-1*β*, IL-6, COX-2, and TNF-*α* in the joint tissues of rats with MIA-induced OA were measured by real-time PCR. # indicates *p* < 0.05 when compared to saline group (NC). *∗* indicates *p* < 0.05 and *∗∗* indicates *p* < 0.01 when compared to the control group (CTL).

**Figure 5:**
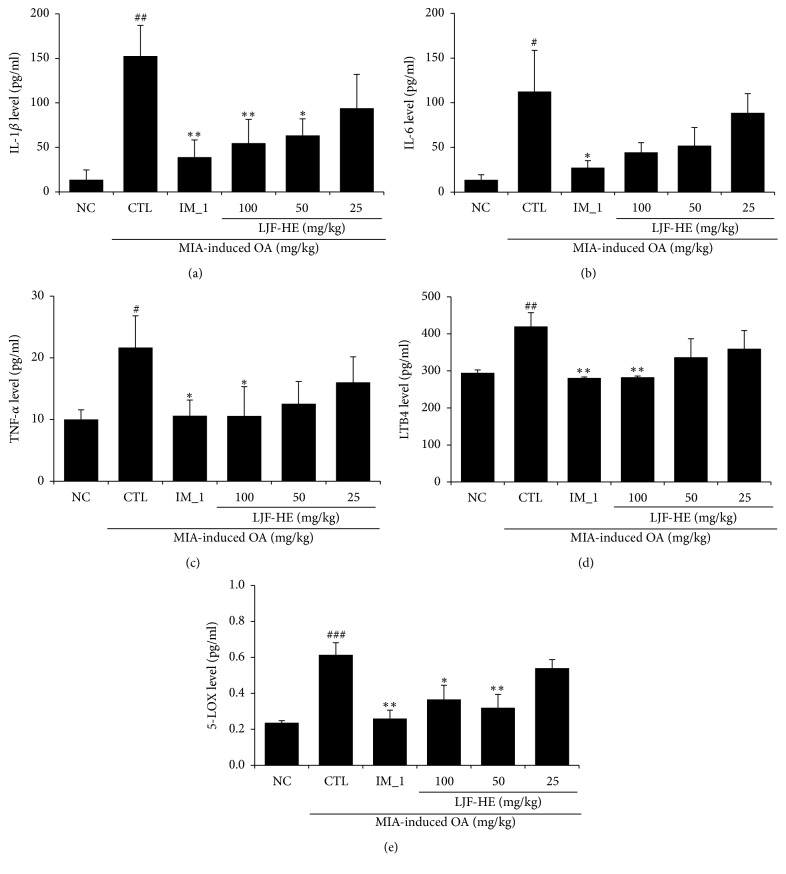
*Effects of LJF-HE on the production of proinflammatory and OA-related pathological factors in the sera of rats with MIA-induced OA*. The serum levels of (a–c) IL-1*β*, IL-6, and TNF-*α*, and (d–e) LTB4 and 5-LOX were measured by ELISA. # indicates *p* < 0.05, ## indicates *p* < 0.01, and ### indicates *p* < 0.001 when compared to NC. *∗* indicates *p* < 0.05 and *∗∗* indicates *p* < 0.01 when compared to the control group (CTL).

Since inflammation is an important factor associated with OA development and progression, we investigated the anti-inflammatory effects of LJF-HE. First, using H&E staining, we observed that the control group showed marked increases in inflammatory cell infiltration in the cartilage and synovial membrane of the MIA-treated joint (Figure 4(a): (B)) compared to that in the healthy joint (Figure 4(a): (A)). However, in the LJF-HE- and IM-treated groups, there was a prominent inhibition of inflammatory cell infiltration and edema (Figure 4(a): (C), (D), and (E)). Next, we examined the mRNA levels of IL-1*β*, IL-6, TNF-*α*, and COX-2 in cartilage and observed that the expression levels of all the cytokines increased in the control group due to the MIA injection. The results also indicated that LJF-HE reduced the expression levels of the cytokines; however, IL-1*β* and TNF-*α* were more affected (*p* < 0.05) than IL-6 and COX-2 were.  Figure 5 shows the serum levels of the various inflammatory markers. MIA induced the production of IL-1*β*, IL-6, and TNF-*α* in serum. Consistent with the above data, the serum levels of IL-1*β* and TNF-*α* were significantly lowered by LJF-HE at a dose of 100 mg/kg. It was also noted that the serum levels of IL-1*β* and TNF-*α* were comparable in the LJF-HE-treated (100 mg/kg) and IM-treated (1 mg/kg) rats (Figures 5(a) and 5(c)). Furthermore, MIA-induced increases in the serum levels of LTB4 and 5-LOX were significantly inhibited by LJF-HE (Figures 5(d) and 5(e)). Taken together, these data suggest that LJH-HE might systemically inhibit inflammation to suppress MIA-induced OA.

### 3.5. In Vitro Effects of LJF-HE on Inflammation

**Figure 6:**
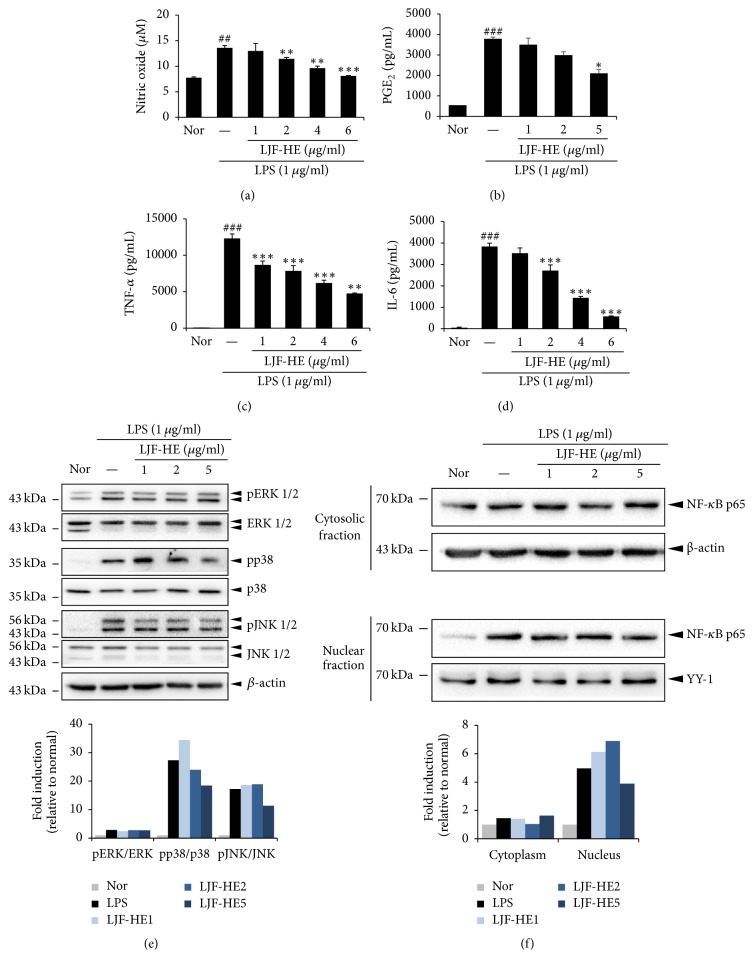
*Effects of LJF-HE on inflammation in LPS-stimulated RAW 264.7 cells*. RAW 264.7 cells were pretreated with LJF-HE (1–6 *μ*g/mL) for 1 h and then treated with LPS (1 *μ*g/mL) for 0.5 h or 18 h. The levels of (a, b) NO and PGE_2_ in the cell culture supernatant were measured using NO and PGE_2_ detection kits. The levels of (c, d) TNF-*α* and IL-6 were measured by ELISA. (e) The phosphorylation levels of ERK1/2, JNK1/2, and p38 were analyzed by western blotting. The relative amount of each protein was determined using ImageJ software. (f) Nuclear, cytosolic, and total proteins were analyzed by western blotting using NF-*κ*B p65 antibodies. *β*-Actin and YY-1 were used as internal controls for the cytosolic and nuclear proteins. The relative amount of each protein was determined using ImageJ software. ## indicates *p* < 0.01 and ### indicates *p* < 0.001 when compared to the untreated cells (Nor). *∗* indicates *p* < 0.05, *∗∗* indicates *p* < 0.01, and *∗∗∗* indicates *p* < 0.001 when compared to the LPS-treated cells.

In order to understand the molecular mechanisms underlying the activity of LJF-HE, we investigated the effects of the extract on LPS-induced inflammatory responses. LJF-HE significantly suppressed the production of various inflammatory mediators and proinflammatory cytokines such as NO, PGE_2_, TNF-*α*, and IL-6 in a dose-dependent manner without affecting cell viability (Figures 6(a)–6(d)). Furthermore, the levels of phosphorylated NF-*κ*B p65 in the nucleus (Figure 6(f)), as well as the phosphorylation of p38 and JNK, were slightly lowered by LJF-HE at 5 *μ*g/mL; however, pERK levels were not significantly reduced (Figure 6(e)). These data suggest that LJF-HE has an anti-inflammatory effect.

## 4. Discussion

Although it is well accepted that OA is a disease characterized by cartilage degeneration, its pathogenesis is not completely understood. During the last decade, accumulated evidences have suggested that mechanical stress-induced inflammatory mechanisms are involved in the development and progression of OA [15]. Inflammation is associated with risks of cartilage loss and disease symptoms such as pain, swelling, stiffness, and synovitis involving inflammatory cell infiltration [16]. Moreover, inflammatory cytokines and mediators are detectable in the synovial fluid in OA [17]. They are usually released from the cartilage and synovial tissue at the affected joint, where chondrocytes and cells in the synovium produce and/or respond to TNF-*α* and IL-1*β* at the early and late stages of OA [18, 19]. Activation of the downstream signaling of these markers induces the synthesis of MMPs and other proteinases by chondrocytes, as well as the production of PGE_2_. These occur via stimulation of COX-2 gene expression or activity [15], as well as via upregulation of the production of NO and other cytokines such as IL-6. NF-*κ*B and MAPK pathways are abnormally activated in OA [20]. In addition, they regulate the expression of genes involved in catabolic pathways [15, 21, 22], which finally leads to cartilage loss and bone deformities.

Several animal models of OA have been developed to study the efficacy of drugs. Chemical injection, surgery, and genetic modification have been used to induce pain and cartilage destruction, as well as changes in the expression levels of several biomarkers in animal models [23, 24]. In the present study, we injected MIA, which is an irreversible inhibitor of nicotinamide adenine dinucleotide phosphate, into the joints of the animals to induce the development of OA. This animal model of OA is relatively easy to induce since only a single injection of MIA is required. In addition, various pathological features that mimic human OA, such as loss of cartilage, synovial proliferation, osteophyte formation, and inflammatory changes are well expressed in the model [25].

Using a rat model of MIA-induced OA, we showed that LJF-HE has potential as an antiosteoarthritic agent. In addition, its effects are mediated via suppression of inflammatory pathways. Results of the measurement of hind paw weight-bearing distribution indicated that LJF-HE protected the joints of the rats from MIA-induced damage. Additionally, histological examination of the joints revealed that proteoglycan degradation and bone resorption were significantly suppressed by LJF-HE. Furthermore, DPD and osteocalcin levels [26] were significantly reduced by LJF-HE. Anti-inflammatory responses were consistently observed in the cartilage, synovial tissues, and serum after treatment with LJF-HE in the rats. Moreover, infiltration of inflammatory cells into damaged cartilage and synovium was prevented. The expression and production of TNF-*α*, IL-1*β*, and IL-6 were significantly reduced by LJF-HE. In addition, LJF-HE suppressed the expression of COX-2 and 5-LOX and the production of PGE_2_, LTB_4_, and NO. It also significantly inhibited the phosphorylation of p38 and JNK.

Considering that several antiosteoarthritic medicines such as COX-2 inhibitors have been withdrawn because of their adverse effects [10], LJF-HE may be an alternative agent for improving joint heath. This is because the results of the present study show that it protects the joints from mechanical stress and inflammation, which induce the development and progression of OA. However, in order to develop LJF-HE as a functional food for improving joint health, the safety and efficacy of the extract and its active components have to be investigated in various animal models and humans.

## Figures and Tables

**Table 1:** Gradient elution method used for the analysis.

Time (min)	% A (0.5% acetic acid)	% B (acetonitrile)
0	40	60
2	40	60
30	0	100
35	0	100
40	40	60
50	40	60

**Table 2:** Real-time PCR primer sequences.

Gene		Primer sequence
IL-1*β*	Forward	5′-CCCTGCAGCTGGAGAGTGTGG-3′
	Reverse	5′-TGTGCTCTGCTTGAGAGGTGCT-3′
IL-6	Forward	5′-TTCCTACCCCAACTTCCAATG-3′
	Reverse	5′-ATGAGTTGGATGGTCTTGGTC-3′
TNF-*α*	Forward	5′-GACCCTCACACTCAGATCATCTTCT-3′
	Reverse	5′-TGCTACGACGTGGGCTACG-3′
COX-2	Forward	5′-TGGTGCCGGGTCTGATGATG-3′
	Reverse	5′-GCAATGCGGTTCTGATACTG-3′
GAPDH	Probe	Applied Biosystems Rat GAPDH Endogenous Control (VIC®/MGB Probe, 4352338E)
